# Hilar GABAergic Interneuron Activity Controls Spatial Learning and Memory Retrieval

**DOI:** 10.1371/journal.pone.0040555

**Published:** 2012-07-05

**Authors:** Yaisa Andrews-Zwilling, Anna K. Gillespie, Alexxai V. Kravitz, Alexandra B. Nelson, Nino Devidze, Iris Lo, Seo Yeon Yoon, Nga Bien-Ly, Karen Ring, Daniel Zwilling, Gregory B. Potter, John L. R. Rubenstein, Anatol C. Kreitzer, Yadong Huang

**Affiliations:** 1 Gladstone Institute of Neurological Disease, San Francisco, California, United States of America; 2 Gladstone Institute of Cardiovascular Disease, San Francisco, California, United States of America; 3 Biomedical Sciences Graduate Program, University of California San Francisco, San Francisco, California, United States of America; 4 Department of Neurology, University of California San Francisco, San Francisco, California, United States of America; 5 Department of Physiology, University of California San Francisco, San Francisco, California, United States of America; 6 Department of Psychiatry, University of California San Francisco, San Francisco, California, United States of America; 7 Department of Pathology, University of California San Francisco, San Francisco, California, United States of America; Massachusetts General Hospital, United States of America

## Abstract

**Background:**

Although extensive research has demonstrated the importance of excitatory granule neurons in the dentate gyrus of the hippocampus in normal learning and memory and in the pathogenesis of amnesia in Alzheimer's disease (AD), the role of hilar GABAergic inhibitory interneurons, which control the granule neuron activity, remains unclear.

**Methodology and Principal Findings:**

We explored the function of hilar GABAergic interneurons in spatial learning and memory by inhibiting their activity through Cre-dependent viral expression of enhanced halorhodopsin (eNpHR3.0)—a light-driven chloride pump. Hilar GABAergic interneuron-specific expression of eNpHR3.0 was achieved by bilaterally injecting adeno-associated virus containing a double-floxed inverted open-reading frame encoding eNpHR3.0 into the hilus of the dentate gyrus of mice expressing Cre recombinase under the control of an enhancer specific for GABAergic interneurons. *In vitro* and *in vivo* illumination with a yellow laser elicited inhibition of hilar GABAergic interneurons and consequent activation of dentate granule neurons, without affecting pyramidal neurons in the CA3 and CA1 regions of the hippocampus. We found that optogenetic inhibition of hilar GABAergic interneuron activity impaired spatial learning and memory retrieval, without affecting memory retention, as determined in the Morris water maze test. Importantly, optogenetic inhibition of hilar GABAergic interneuron activity did not alter short-term working memory, motor coordination, or exploratory activity.

**Conclusions and Significance:**

Our findings establish a critical role for hilar GABAergic interneuron activity in controlling spatial learning and memory retrieval and provide evidence for the potential contribution of GABAergic interneuron impairment to the pathogenesis of amnesia in AD.

## Introduction

The hippocampus plays a key role in spatial learning and memory and is one of the most vulnerable regions of the brain to Alzheimer's disease (AD) pathology [1], [2]. The dentate gyrus is the gateway to the hippocampus and receives synaptic inputs from the entorhinal cortex [1]. The dentate gyrus consists of >95% excitatory granule neurons and <5% inhibitory GABAergic interneurons concentrated in the hilus [3]. Hilar interneurons prevent overexcitation of granule neurons and participate in the formation and regulation of brain oscillations [4], [5]. The balance of excitatory and inhibitory neuronal activity in the hippocampus, including the dentate gyrus, is thought to be required for normal learning and memory [6], while an imbalance has been implicated in the pathogenesis of amnesia in Alzheimer's disease (AD) and schizophrenia [7], [8], [9], [10]. Although extensive research has demonstrated the importance of excitatory granule neurons in learning and memory [8], the role of hilar GABAergic interneurons remains unclear. The current study was designed to explore the function of hilar GABAergic interneurons in spatial learning and memory by optogenetically inhibiting their activities during cognitive tests.

## Materials and Methods

### Mice

Hemizygous Dlx-I12b-Cre (I12b-Cre) mice expressing Cre recombinase under the control of an enhancer specific for forebrain GABAergic interneurons [11] were bred with wildtype mice to generate I12b-Cre and wildtype littermates. Hemizygous I12b-Cre mice were also bred with Lhx6-GFP BAC transgenic mice, in which GFP was expressed specifically in GABAergic interneurons [12], to generate mice expressing both Cre and GFP in GABAergic interneurons. All mice were on a C57BL/6 genetic background and used at 7–9 weeks of age (I12b-Cre/Lhx6-GFP mice for *in vitro* electrophysiological studies) or 3–5 months of age (I12b-Cre and wildtype mice for *in vivo* electrophysiological and behavioral studies). All procedures were approved by the Gladstone Institutes and the University of California San Francisco Animal Care and Use Committees.

### Viral expression of DIO-eNpHR3.0-eYFP and DIO-eYFP in I12b-Cre transgenic mice

Bilateral injections of recombinant AAV1-DIO-eNpHR3.0-eYFP or AAV1-DIO-eYFP virus targeted the hilus of the dentate gyrus of the hippocampus in I12b-Cre transgenic mice. The DIO constructs virtually eliminate recombination in cells that do not express Cre recombinase [13]. The double-floxed inverted eNpHR3.0-eYFP or eYFP cassette was cloned into a modified version of the pAAV2-MCS vector (Stratagene) carrying the EF-1-alpha promoter and the WPRE to enhance expression. The recombinant AAV vectors were serotyped with AAV1 coat proteins and packaged by the viral vector core at the University of North Carolina. The final viral concentration was 2×10^12^ viral particles per milliliter (by Dot Blot, UNC vector core).

### Stereotaxic viral injections

Anesthesia was induced with a mixture of ketamine and xylazine (100 mg ketamine plus 5 mg xylazine per kilogram of body weight) through intraperitoneal injection and maintained with 1% isoflurane through a nose cone mounted on a stereotaxic apparatus (Kopf Instruments).

For mice used in slice recordings, the scalp was opened and two holes were drilled in the skull (2.1 mm AP, 1.5 mm ML from bregma). DIO-eNpHR3.0-eYFP virus (1 µl per side) was injected bilaterally into the hilus of the dentate gyrus of the hippocampus (2.1 mm DV from top of skull) through a 33-gauge steel injection cannula (PlasticsOne) with a syringe pump (World Precision Instruments) that infused the virus over 10 min. The injection cannula was left in place for 5–10 min after the injection and then slowly removed.

For the mice that were used in behavioral experiments and *in vivo* recordings, the scalp was opened and two holes were drilled in the skull. The mice were then implanted with bilateral guide cannulae (26-gauge, 2.1 mm deep, measured from top of skull; PlasticsOne) aimed at the following coordinates: 2.1 mm AP, 1.5 mm ML from bregma. After implantation of the guide cannula, viral injections (DIO-eNpHR3.0-eYFP or DIO-eYFP) were made through a 33-gauge steel injection cannula (PlasticsOne) that was passed through the guide cannula, such that the tip of the injection cannula extended 0.05 mm from the end of the guide cannula. DIO-eNpHR3.0-eYFP or DIO-eYFP virus (1 µl per side) was injected bilaterally into the hilus (2.1 mm DV from top of skull) through the steel injection cannula with a syringe pump (World Precision Instruments) that infused the virus over 10 min. The injection cannula was left in place for 5–10 min after the injection and then slowly removed. After the injections, dummy cannulae (PlasticsOne) were inserted into the guide cannulae to maintain patency. All surgical procedures were performed under aseptic conditions. To allow time for recovery and viral expression, animals were housed for at least 3–4 weeks after injection and before any recording or behavioral experiments were initiated.

### Immunohistochemistry and image collection

Brain tissues from 4–6-month-old mice after behavioral tests were collected after a 1-minute transcardial perfusion with 0.9% NaCl. The whole brain from each mouse was fixed in 4% paraformaldehyde. Sliding microtome sections (30 µm) were immunostained with the following primary antibodies: polyclonal goat anti-GFP (1∶2000 for fluorescence; AbCam), mouse anti-NeuN (1∶200 for fluorescence; Chemicon), rabbit anti-GABA (1∶3000 for fluorescence; Sigma), rabbit anti-cFos (1∶2000, for fluorescence; Calbiochem). Primary antibodies were detected with fluorescein-labeled donkey anti-rabbit IgG (1∶500; Invitrogen), Alexa Fluor 594–labeled donkey anti-mouse IgG (1∶2000; Invitrogen), or Cy5–labeled anti-mouse IgG (1∶500; Jackson ImmunoResearch). Stained sections were examined with a Radiance 2000 laser-scanning confocal system (Bio-Rad) mounted on a Nikon Optiphot-2 microscope or a Leica microscope [7]. cFos-positive cells were quantified in the dentate gyrus, the CA3, and the CA1 regions of the hippocampus on immunostained sections. GABA and eNpHR3.0-eYFP double positive cells were quantified in the hilus of the dentate gyrus, the CA3, and the CA1 regions of the hippocampus. These were quantified by counting their numbers in every tenth serial coronal section throughout the rostrocaudal extent of the hippocampi by an investigator blinded to genotype and treatment [7]. The number of counted cells for each immunostaining was multiplied by 10 (for every tenth serial section) to obtain the total number of the immunostained cells per hippocampi. The percentage of eNpHR3.0-eYFP-positive cells also positive for GABA, and the percentage of GABA-positive cells also positive for eNpHR3.0-eYFP were calculated from brain sections doubly stained for GABA and eNpHR3.0-eYFP.

### Optical stimulation and behavioral analysis in awake mice

Two glass fibers (AFS105/125Y, Thorlabs) were connected with SMA connectors at one end and cleaved flat at the other end, and were inserted through guide cannulae into the hilus of the hippocampus. The 594-nm-laser power was adjusted to yield 1 mW intensity at the tip of each fiber (measured with a PM100D optical power meter with an S120C sensor, Thorlabs). The positions of the nose, tail and center of mass of each mouse were tracked by Noldus (Netherlands), a video tracking system, during each trial of the following behavioral tests.

### Morris water maze test

The water maze consisted of a pool (121 cm diameter) filled with water (21±1°C) made opaque with non-toxic white tempera paint powder; the pool was located in a room surrounded by distinct extra-maze (spatial) cues [7]. Before hidden platform training, mice were given four pre-training trials in which they had to swim in a rectangular channel (15 cm×122 cm) and mount a platform hidden 1.5 cm below the water surface in the middle of the channel. Mice that did not mount the platform were gently guided to it and were allowed to sit for 10 seconds, after which they were rescued by the experimenter. The maximum latency per trial in this task was 90 seconds.

The day after pre-training, mice were trained in the circular water maze. For hidden platform training, the platform (14 cm×14 cm) was submerged 1.5 cm below the water surface. The platform location remained the same throughout hidden platform training, but the drop location varied semi-randomly between trials. Mice received two training sessions with 3-hour inter-session intervals for five consecutive days. Each session consisted of two trials with 10-minute inter-trial intervals. The maximum latency per trial in this task was 60 seconds. If a mouse did not find the platform, it was guided to it and allowed to sit on it for 10 seconds.

In spatial probe trials, the platform was removed, and mice were allowed to swim for 60 seconds before rescue. The drop location was 180° from where the platform was placed during hidden platform training. Drop location remained constant throughout all spatial probe trials.

After the final spatial probe, mice were given one day of rest and then visible platform training was performed. In this task, the platform had an additional visible cue (15 cm tall black and white striped pole) placed on top of the platform. Mice received two training sessions per day with 3–4-hour inter-trial intervals. Each session consisted of two training trials with 10-minute inter-trial intervals. The maximum latency per trial in this task was 60 seconds. For each session, the platform was moved to a new location and the drop location varied semi-randomly between trials.

The hilus of the hippocampi of some behaving mice were bilaterally illuminated (594 nm) with 1 mW intensity for the duration (60 s) of the various tasks (hidden training, spatial probes, and visible training). Behavior was recorded with a video tracking system. Escape latencies, swim paths, swim speeds, percent time spent in each quadrant, and platform crossings were recorded for subsequent data analyses.

### Y-maze test

The apparatus consisted of three symmetrical arms in the shape of a Y [14]. Before testing, mice were transferred to the testing room and acclimated for at least 1 hour. During testing, each mouse was gently placed in a starting arm facing the wall and arm entries were recorded for 6 minutes, divided into six 1-minute intervals. The maze was cleaned with 70% alcohol between testing of each mouse. The hilus of the hippocampi of some behaving mice were bilaterally illuminated (594 nm) with 1 mW intensity for the duration of the tasks. Spontaneous alternations and total activities were calculated.

### Rotarod test

Motor coordination and balance were evaluated using the rotarod (Med Associates, St. Albans, VT) [14]. Mice were placed on the apparatus with the rod rotating at the speed of 16 (day 1) and 4–40 (subsequent days) rotations per minute (RPM) for three trials. Each trial was complete when the mouse fell down or when 5 minutes elapsed (whichever came first). The inter-trial interval for each mouse was 15 min. The apparatus was cleaned with 70% alcohol between testing of each mouse. The hilus of the hippocampi of some behaving mice were bilaterally illuminated (594 nm) with 1 mW intensity for the duration of the tasks. Latency to fall was recorded for subsequent data analyses.

### Open field test

Spontaneous locomotor activity in an open field was measured in an automated Flex-Field/Open Field Photobeam Activity System (San Diego Instruments, San Diego, CA) [14]. Before testing, mice were transferred to the testing room and were acclimated for at least 1 hour. Mice were tested in a clear plastic chamber (41×41×30 cm) for 15 min, with two 16×16 photobeam arrays detecting horizontal and vertical movements. The apparatus was cleaned with 70% alcohol between testing of each mouse. The hilus of the hippocampi of some behaving mice were bilaterally illuminated (594 nm) with 1 mW intensity for the duration of the tasks. Total movements (ambulations) in the outer periphery and center of the open field were recorded for further data analyses.

### Slice recordings

Coronal sections (300 µm thick) containing hippocampi were prepared from brains of 7–9 week-old I12b-Cre mice expressing eNpHR3.0-eYFP in GABAergic interneurons. The young mice were used solely for verifying the presence of meaningful halorhodopsin currents in hilar interneurons. Since this particular mouse line expresses Cre in many different types of GABAergic interneurons [11], we did not attempt to sample all the different subtypes of interneurons found in the hilus, nor to identify the specific subtypes we recorded. The mice had been bilaterally injected at 3 weeks of age with AAV1-DIO-eNpHR3.0-eYFP virus. Slices were prepared using a vibratome (Leica VT1000S) in ice-cold carbogenated N-methyl-D-glucamine (NMDG) solution containing (in mM) 135 NMDG, 1 KCl, 1.2 KH_2_PO_4_, 1.5 MgCl_2_, 0.5 CaCl_2_, 20 choline HCO_3_, and 12.5 glucose. Slices were subsequently transferred to a holding chamber containing artificial cerebrospinal fluid (ACSF) containing (in mM) 125 NaCl, 26 NaHCO_3_, 1.25 NaH_2_PO_4_.H_2_O, 2.5 KCl, 1 MgCl, 2 CaCl, and 12.5 glucose at 33°C for 30 min. After resting for 1–5 h at room temperature, experiments were performed on slices perfused with ACSF, warmed to 31–33°C [15].

Hippocampal slices were visualized using an Olympus BX51WI microscope equipped with epifluorescence. YFP-positive neurons were selected for whole-cell recording. For characterization of halorhodopsin-mediated responses, the internal solution contained (in mM) 130 KMeSO_3_, 10 NaCl, 2 MgCl_2_, 0.16 CaCl_2_, 0.5 EGTA, 10 HEPES, 2 Mg-ATP, and 0.3 Na-GTP, pH 7.3. All recorded neurons exhibited electrophysiological characteristics of hilar interneurons, and likely represented multiple interneuron subtypes.

Excitation of eNpHR3.0 was achieved by epifluorescence (100-W mercury arc lamp, excitation filter; Chroma ET560-630/40×) and gated by a Uniblitz VS25 shutter (Vincent Associates) under through-the-lens control. Measured light intensity at the slice was approximately 1 mW cm^−2^. Data were collected with a MultiClamp 700B amplifier (Molecular Devices) and ITC-18 A/D board (HEKA) using IGOR PRO 6.0 software (Wavemetrics) and custom acquisition routines (mafPC, courtesy of M. A. Xu-Friedman). Current-clamp recordings were filtered at 10 kHz and digitized at 40 kHz. Electrodes were made from borosilicate glass (pipette resistance, 2–4 MΩ). The hyperpolarization induced by yellow light was calculated as the difference between the average membrane potential at rest during the 100 ms prior to the light pulse and the average nadir of the membrane potential during 100 ms light pulses. To assess the ability of yellow light to stop the firing of action potentials, cells were depolarized to spike with direct current injection, then pulsed with yellow light.

### Anaesthetized hippocampal optrode recordings *in vivo*

After the behavioral tests were completed, eNpHR3.0-eYFP-positive mice were anaesthetized with a mixture of ketamine and xylazine (100 mg ketamine plus 5 mg xylazine per kilogram of body weight i.p.) and maintained with both isoflurane and ketamine/xylazine injections. The scalp of the animal was opened and the craniotomy that was used for the viral injection was cleaned out and expanded with a surgical drill. The optrode was then lowered through this craniotomy. We coupled the silicon optrode to a 594-nm laser (OEM laser systems) via a fiber-optic patch cord, and used the optrode to record dentate granule neuron activity in anaesthetized eNpHR3.0-eYFP-positive mice [15].

After each recording, the probe was lowered or moved to a new recording tract such that multiple recordings were made from each mouse. The 594-nm-laser power was 1 mW at the tip of the optical fiber for all optrode recordings (measured with a PM100D optical power meter with an S120C sensor, Thorlabs).

### Analysis of anaesthetized recordings

Voltage signals from each recording site on the silicon probe were band-pass-filtered, such that activity between 0.7 and 300 Hz was analyzed as LFPs and activity between 150 and 8,000 Hz was analyzed as spiking activity [15]. Both types of data were amplified, processed and digitally captured using commercial hardware and software (Plexon). Single units were discriminated with principal component analysis (OFFLINE SORTER 3.0.1, Plexon). Two criteria were used to ensure quality of recorded units: (1) recorded units smaller than 100 µV (∼3 times the noise band) were excluded from further analysis and (2) recorded units in which more than 1% of interspike intervals were shorter than 2 ms were excluded from further analysis. We tested each recorded neuron for a significant increase in firing rate during the entire period when the laser was on (2–5 s), relative to an identical time period directly preceding the laser illumination (paired *t*-tests across all laser presentations).

### Statistical Analyses

Values are expressed as mean ± SEM. Statistical analyses were performed with GraphPad Prism. Differences between means were assessed by *t*-test, one-factor ANOVA, or repeated measures ANOVA, followed by Bonferroni or Tukey-Kramer post hoc tests. *P*<0.05 was considered statistically significant.

## Results

### Selective virus-mediated eNpHR3.0 expression in hilar GABAergic interneurons

**Figure 1:**
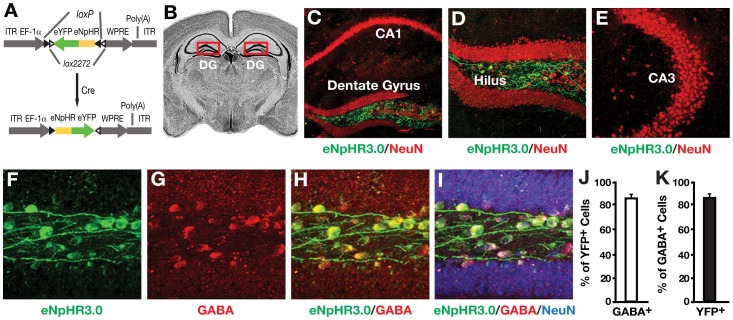
Selective virus-mediated eNpHR3.0 expression in hilar GABAergic interneurons. (A) Schematic of the double-floxed Cre-dependent AAV1 vector expressing eNpHR3.0-eYFP under control of the EF-1-alpha promoter. eYFP, enhanced yellow fluorescent protein; ITR, inverted terminal repeat; WPRE, woodchuck hepatitis virus posttranscriptional regulatory element. (B) Coronal mouse brain section. Red box indicates the dentate gyrus (DG) of the hippocampus. (C–E) Confocal images of the DG and CA1 (C), the hilus (D), and the CA3 (E) regions of the hippocampus of I12b-Cre mice injected with AAV1-DIO-eNpHR3.0-eYFP virus. Green indicates the expression of eNpHR3.0-eYFP; red indicates neurons stained positive for NeuN. (F–I) Confocal images of hilar cells expressing eNpHR3.0-eYFP (green), GABA (red), or NeuN (blue) in I12b-Cre mice injected with AAV1-DIO-eNpHR3.0-eYFP virus. Yellow indicates the colocalization of eNpHR3.0-eYFP and GABA (H). (J) Percent of eYFP-positive hilar cells also positive for GABA. (K) Percent of GABA-positive hilar cells also positive for eYFP. Values are mean ± SEM (n = 6 mice).

To explore the function of hilar GABAergic interneurons in spatial learning and memory, we tried to obtain selective optogenetic control of hilar GABAergic interneuron activity *in vivo* by bilaterally injecting adeno-associated virus (AAV1) containing a double-floxed inverted open-reading frame encoding a fusion of eNpHR3.0 and enhanced yellow fluorescent protein (DIO-eNpHR3.0-eYFP) [16] (Fig. 1*A*). The virus was injected bilaterally into the hilus of the dentate gyrus (Fig. 1*B*) of mice expressing Cre recombinase under the control of the Dlx-I12b enhancer specific for forebrain GABAergic neurons (I12b-Cre line), in which nearly all hilar GABAergic interneurons express Cre [11]. eNpHR3.0-eYFP transcription is enabled only in cells producing Cre (Fig. 1*A*), thus restricting expression to GABAergic interneurons [13]. Confocal imaging of coronal sections of injected mouse brains revealed numerous eNpHR3.0-eYFP-positive cells in the hilus but not in the CA1 or CA3 region of the hippocampus (Figs. 1*C–E*). Most eNpHR3.0-eYFP-positive hilar cells (∼85%) were positive for GABA (a GABAergic interneuron marker) (Figs. 1*F–J*), confirming that they were GABAergic interneurons. Furthermore, >85% of GABA-positive hilar interneurons were positive for eNpHR3.0-eYFP (Fig. 1*K*), suggesting effective expression of eNpHR3.0-eYFP in hilar GABAergic interneurons. In contrast, <15% of GABAergic interneurons in the CA1 or CA3 regions expressed eNpHR3.0-eYFP. Importantly, dentate granule neurons and CA1 and CA3 pyramidal neurons were eNpHR3.0-eYFP-negative (Figs. 1*C–E,I*). Wildtype I12b-Cre littermates receiving hilar injection of AAV1-DIO-eNpHR3.0-eYFP virus showed no eNpHR3.0-eYFP expression.

### *In vitro* and *in vivo* light-elicited inhibition of hilar GABAergic interneurons and activation of dentate granule neurons

**Figure 2:**
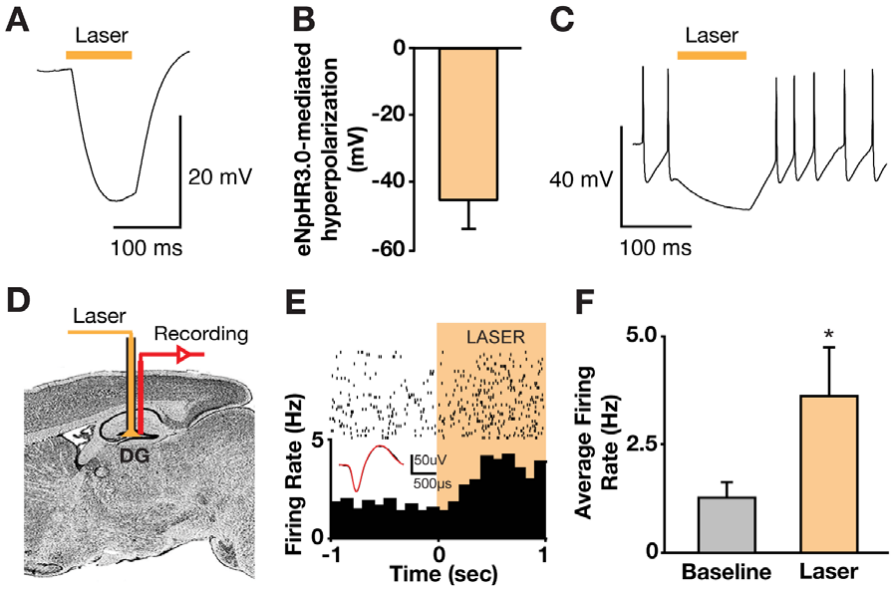
Light-elicited inhibition of hilar GABAergic interneurons and activation of dentate granule neurons. (A, B) Example trace (A) and summary graph (B, n = 5) of eNpHR3.0-mediated membrane hyperpolarization of hilar GABAergic interneurons in brain slices. In this and subsequent panels, yellow bars indicate illumination time. (C) eNpHR3.0-mediated inhibition of spiking of hilar GABAergic interneurons in brain slices. (D) Schematic of *in vivo* optical stimulation and recording in the dentate gyrus (DG) of I12b-Cre mice injected with AAV1-DIO-eNpHR3.0-eYFP virus. (E) Example of a granule neuron recorded from the DG of an anaesthetized I12b-Cre mouse injected with AAV1-DIO-eNpHR3.0-eYFP that showed increased firing in response to yellow laser illumination. Inset shows spike waveform with laser illumination. (F) Average change in dentate granule neuron firing rates in response to yellow laser illumination in I12b-Cre mice injected with AAV1-DIO-eNpHR3.0-eYFP virus. Values are mean ± SEM (n = 7, *p<0.05, two-tailed and unpaired *t*-test).

To test eNpHR3.0 function, we performed whole-cell patch-clamp recordings in brain slices prepared from AAV1-DIO-eNpHR3.0-eYFP-injected I12b-Cre mice. Yellow light illumination (594 nm) of eNpHR3.0-eYFP-positive hilar GABAergic interneurons elicited large light-evoked membrane hyperpolarization (Figs. 2*A,B*) and inhibition of spiking (Fig. 2*C*), indicating that eNpHR3.0 was functional in hilar GABAergic interneurons. We then determined the *in vivo* effect of optogenetic inhibition of hilar GABAergic interneuron activity on the firing pattern of dentate granule neurons in mice injected with AAV1-DIO-eNpHR3.0-eYFP virus. *In vivo* dentate gyrus recordings (Fig. 2*D*) were performed with an optrode (a linear, 16-site silicon electrode array with an integrated laser-coupled optical fiber) that elicited light-induced neuronal activity changes up to 800 µm from the fiber tip [15]. Searching for neurons that responded to yellow laser illumination (1 mW at fiber tip), we identified seven with increased firing during the laser pulse, with an average change from 1.2±0.4 to 3.5±1.2 Hz (*P*<0.05) (Figs. 2*E,F*). Their baseline firing rate (1.2±0.4 Hz, Fig. 2*F*) and waveform duration (Fig. 2*E*) agreed with those of dentate granule neurons in live mice [17], [18]. Importantly, the elevated firing rate returned to baseline within 1.5 seconds after illumination was terminated, suggesting that the precise optogenetic inhibition of hilar interneurons resulted in transient disinhibition of dentate granule neurons.

**Figure 3:**
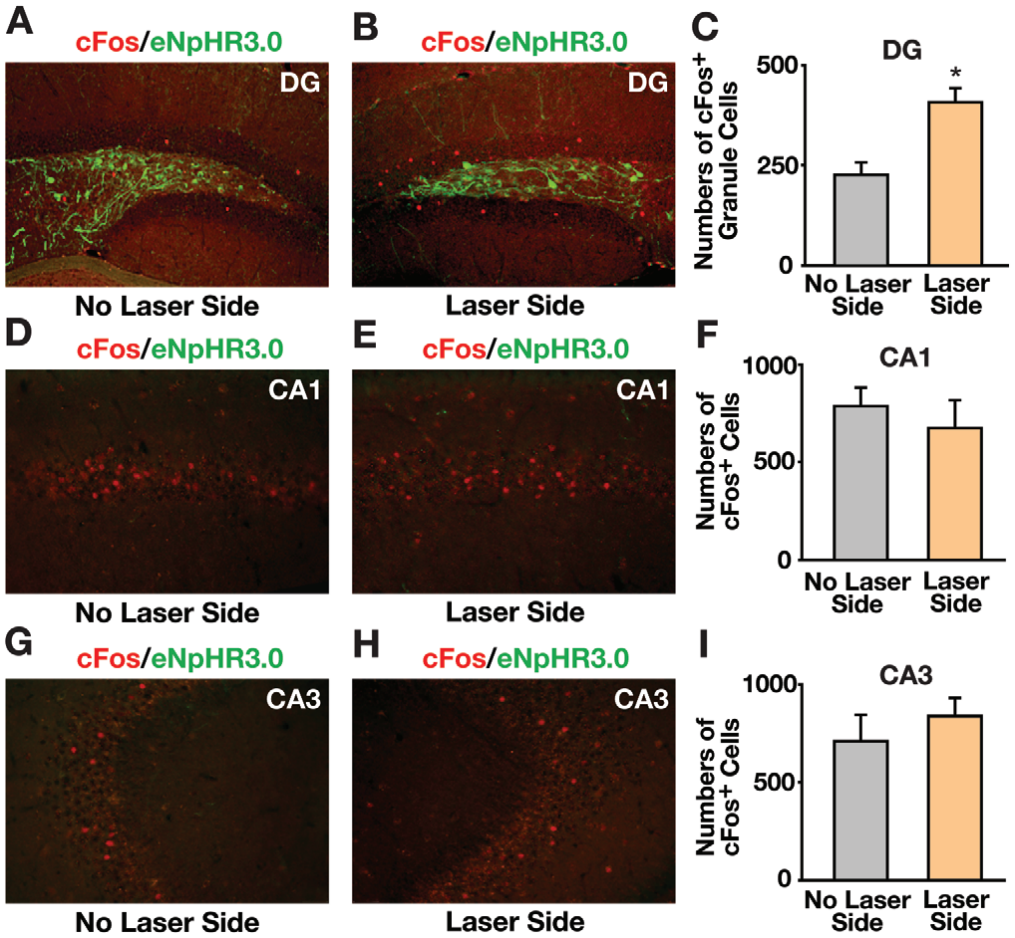
Light-elicited inhibition of hilar GABAergic interneurons significantly increased cFos-positive neurons in the dentate gyrus. (A, B) Confocal images of neurons positive for cFos (red) and eNpHR3.0-eYFP (green) on the side of DG without (A) or with (B) laser illumination in I12b-Cre mice injected with AAV1-DIO-eNpHR3.0-eYFP virus. (C) Quantification of cFos-positive dentate granule neurons on the side of DG without or with laser illumination in I12b-Cre mice injected with AAV1-DIO-eNpHR3.0-eYFP virus. Values are mean ± SEM (n = 6, *p<0.05, two tailed and paired *t*-test). (D, E, G, H) Confocal images of CA1 (D, E) and CA3 (G, H) neurons positive for cFos (red) and eNpHR3.0-eYFP (green) on the side of the hippocampus without (D, G) or with (E, H) laser illumination in the hilus of the dentate gyrus in I12b-Cre mice injected with AAV1-DIO-eNpHR3.0-eYFP virus. (F, I) Quantification of cFos-positive CA1 (F) and CA3 (I) neurons on the side of the hippocampus without or with laser illumination in the hilus of the dentate gyrus in I12b-Cre mice injected with AAV1-DIO-eNpHR3.0-eYFP virus. Values are mean ± SEM (n = 6 mice).

The number of cFos-positive dentate granule neurons, which reflects neuronal activation [15], [19], was also significantly higher on the illuminated side of the dentate gyrus compared to the contralateral, non-illuminated dentate gyrus (*P*<0.05) (Figs. 3*A–C*). However, laser illumination in the hilus did not alter the number of cFos-positive neurons in the CA1 (Fig. 3*D–F*) and CA3 regions (Figs. 3*G–I*). Thus, based on the recordings and cFos data, we obtained precise optogenetic control of hilar GABAergic interneuron activity and, consequently, dentate granule neuron activity in live mice.

### *In vivo* inhibition of hilar GABAergic interneuron activity impaired spatial learning and memory

**Figure 4:**
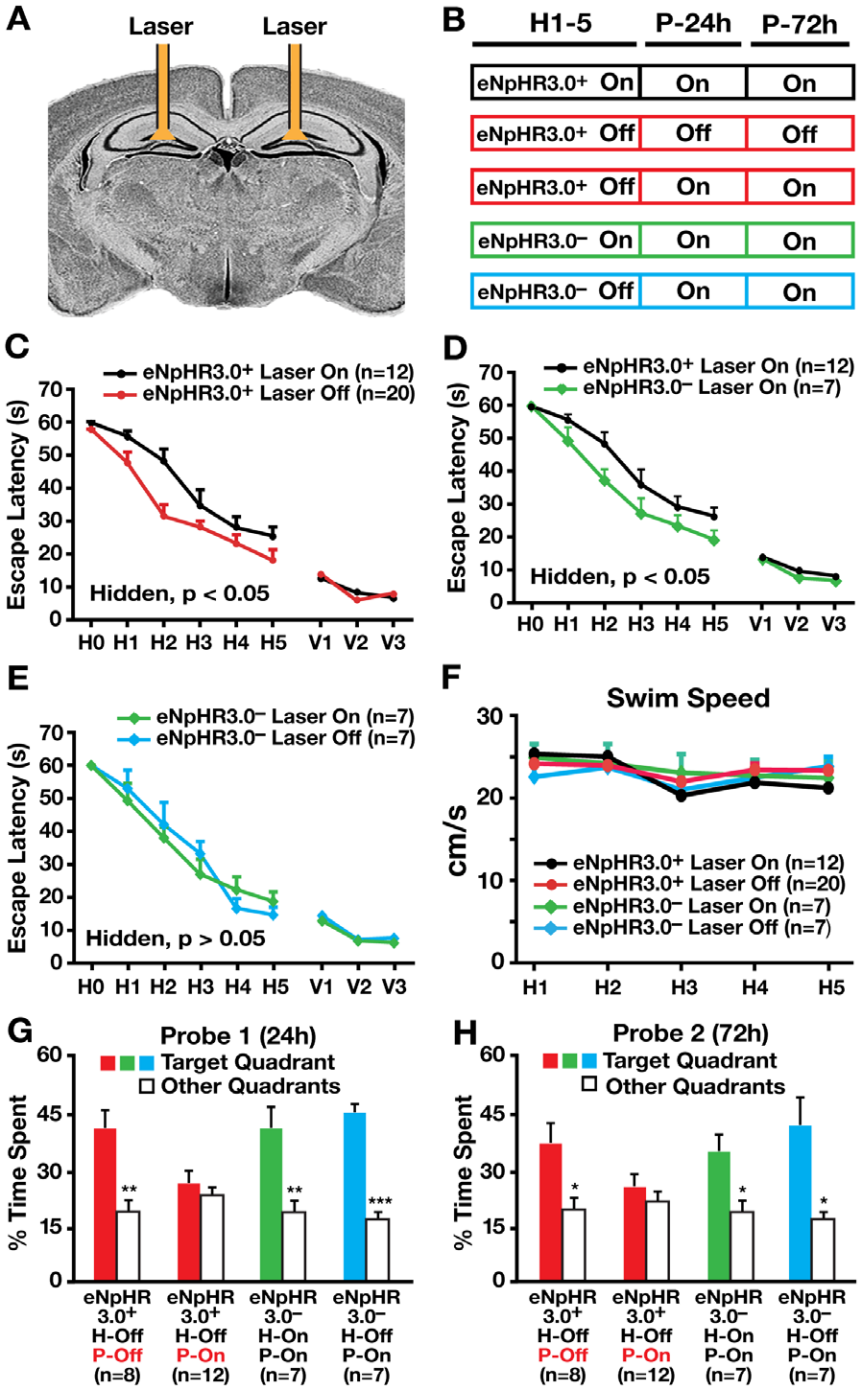
Inhibition of hilar GABAergic interneuron activity impaired spatial learning and memory. (A) Coronal schematic of cannula placement and bilateral fiber-optic stimulation. (B) Protocols of mice used and laser illumination during hidden platform (H1–5) and probe (P-24 h and P-72 h) trials in the Morris water maze (MWM) test. (C–E) Learning curves of I12b-Cre (eNpHR3.0^+^) and wildtype (eNpHR3.0^−^) littermates injected with AAV1-DIO-eNpHR3.0-eYFP virus, with or without laser illumination in MWM tests. Points represent averages of daily trials. H, hidden platform sessions (two trials/session, two sessions/day); H0, first trial on H1; V, visible platform sessions (two trials/session, two sessions/day). Y-axis indicates time to reach the target platform (escape latency). Values are mean ± SEM and statistically evaluated by repeated measures ANOVA. (F) Swim speed did not differ significantly among different groups of mice during the MWM test. (G) Percent time spent in the target quadrant versus the other quadrants in the probe trial performed 24 h (probe 1) after the last hidden platform trial. (H) Percent time spent in the target quadrant versus the other quadrants in the probe trial performed 72 h (probe 2) after the last hidden platform trial. Values are mean ± SEM. n = 7–20 mice/group. *p<0.05 **p<0.01, ***p<0.005 (two tailed and unpaired *t*-test).

**Figure 5:**
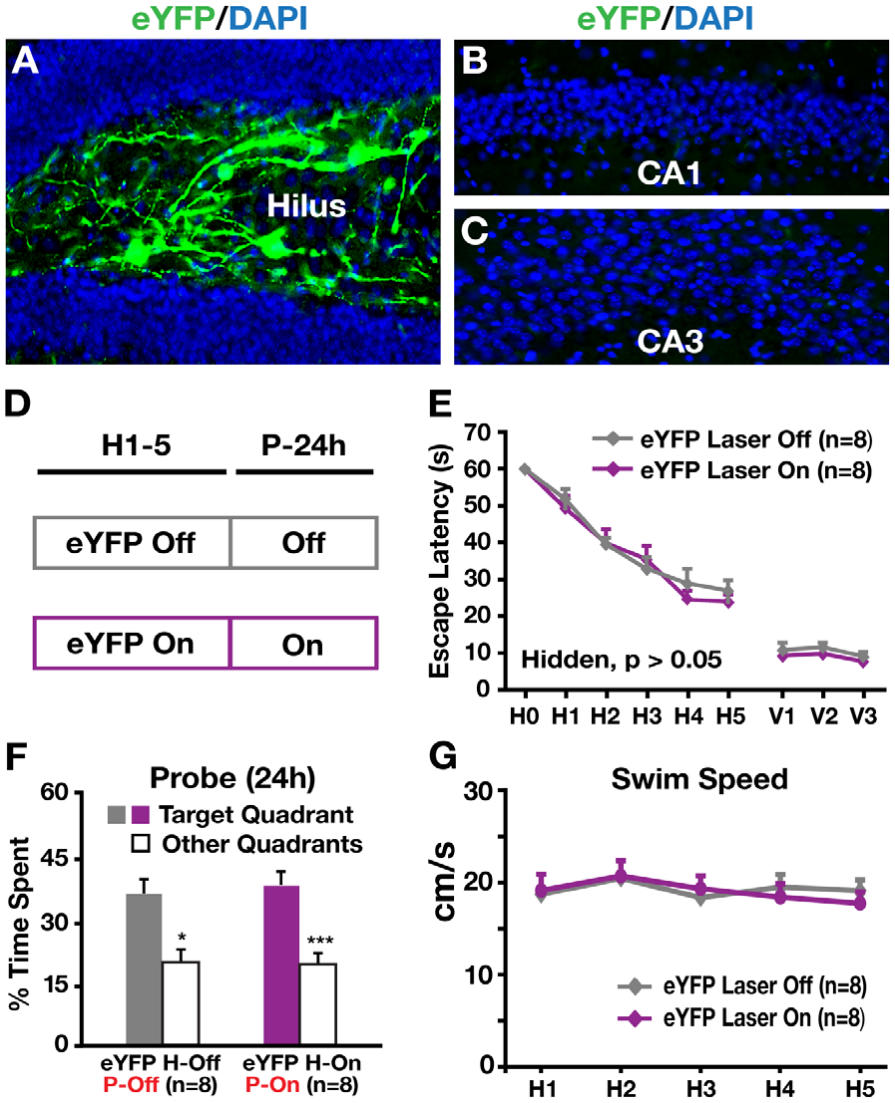
Hilar illumination did not alter learning and memory in I12b-Cre mice injected with eYFP virus. (A–C) Confocal images of the dentate gyrus (A), the CA1 (B), and the CA3 (C) regions of the hippocampus of I12b-Cre mice injected with AAV1-DIO-eYFP virus. Green indicates the expression of eYFP; blue indicates cell nuclei stained positive for DAPI. (D) Protocols of mice used and laser illumination during hidden platform and probe trials in the Morris water maze (MWM) test. (E) Learning curves of AAV1-DIO-eYFP virus-injected I12b-Cre mice with (On) or without (Off) laser illumination did not differ in both hidden and visible platform trials of the MWM test. Points represent averages of daily trials. H, hidden platform sessions (two trials/session, two sessions/day); H0, first trial on H1. Y-axis indicates time to reach the target platform (escape latency). Values are mean ± SEM. (F) Percent time spent in the target quadrant versus the other quadrants in the probe trial performed 24 h after the last hidden platform trial with (P-On) or without (P-Off) laser illumination. Values are mean ± SEM. *p<0.05, ***p<0.005 (two-tailed and unpaired *t*-test). (G) Swim speed did not differ between the two groups of mice during the MWM test. Values are mean ± SEM. n = 8 mice/group.

We next assessed the effect of inhibiting hilar GABAergic interneuron activity on spatial learning and memory in behaving mice in the Morris water maze (MWM). Cannulae were surgically implanted bilaterally into the hilus and used to guide viral injections and fiber-optic placements (Fig. 4*A*) [15]. I12b-Cre mice receiving hilar injection of AAV1-DIO-eNpHR3.0-eYFP were divided into two groups: one with laser illumination (eNpHR3.0^+^ On) and one without (eNpHR3.0^+^ Off) during each 60-s hidden and visible platform trial (Fig. 4*B*). As controls, wildtype I12b-Cre littermates receiving hilar injection of AAV1-DIO-eNpHR3.0-eYFP, in which eNpHR3.0-eYFP was not expressed, were divided into two groups: with (eNpHR3.0^−^ On) and without (eNpHR3.0^−^ Off) laser illumination (Fig. 4*B*). For non-illuminated mice, laser-disconnected optical fibers were inserted into the cannulae during behavioral testing to control for the procedure. Bilateral laser illumination of the hilus, which inhibits hilar GABAergic interneuron activity and consequently increases dentate granule cell activity, elicited learning impairment of eNpHR3.0^+^ mice compared to eNpHR3.0^+^ mice without illumination (Fig. 4*C*), suggesting that inhibiting hilar GABAergic interneuron activity impairs spatial learning. Illuminated eNpHR3.0^+^ mice also showed impaired learning compared to illuminated eNpHR3.0^−^ mice (Fig. 4*D*), suggesting that the impairment was not due to illumination alone. eNpHR3.0^−^ mice did not differ in learning with or without illumination (Fig. 4*E*), also suggesting that the injection and illumination procedures did not affect learning. In visible platform trials, all mice with or without illumination performed well (Figs. 4*C–E*). Swim speed did not differ among the mice (Fig. 4*F*). Importantly, bilateral illumination of I12b-Cre mice receiving hilar injection of AAV1-DIO-eYFP virus as controls, in which eYFP was expressed in hilar GABAergic interneurons (Figs. 5*A–C*), did not impair learning during hidden platform trials and had no effects on visible platform trials and swimming speeds (Figs. 5*D,E,G*), suggesting that the injection and illumination of eYFP did not alter spatial learning.

### *In vivo* inhibition of hilar GABAergic interneuron activity impaired spatial memory retrieval but not memory retention

To assess the effect of inhibiting hilar GABAergic interneuron activity on spatial memory, 24 (probe 1) and 72 (probe 2) hours after the last hidden platform trial, a 60-s probe trial (platform removed) was performed for different groups of mice with or without illumination (Fig. 4*B*). Bilateral laser illumination during probe trials of eNpHR3.0^+^ mice (P-On), which did not receive illumination and learned normally during the hidden platform trials (H-Off), impaired spatial memory in probe 1 (Fig. 4*G*) and probe 2 (Fig. 4*H*) trials, suggesting that inhibiting hilar GABAergic interneuron activity impairs spatial memory. Illuminating eNpHR3.0^−^ mice did not alter spatial memory in both probe trials (Figs. 4*G,H*), regardless of illumination during the hidden platform trials, suggesting that the injection and illumination did not alter spatial memory. Furthermore, bilateral illumination of I12b-Cre mice receiving hilar injection of AAV1-DIO-eYFP virus as controls, in which eYFP was expressed in hilar GABAergic interneurons (Figs. 5*A–C*), did not impair spatial memory (Fig. 5*F*), suggesting that the injection and illumination of eYFP did not confound the results.

**Figure 6:**
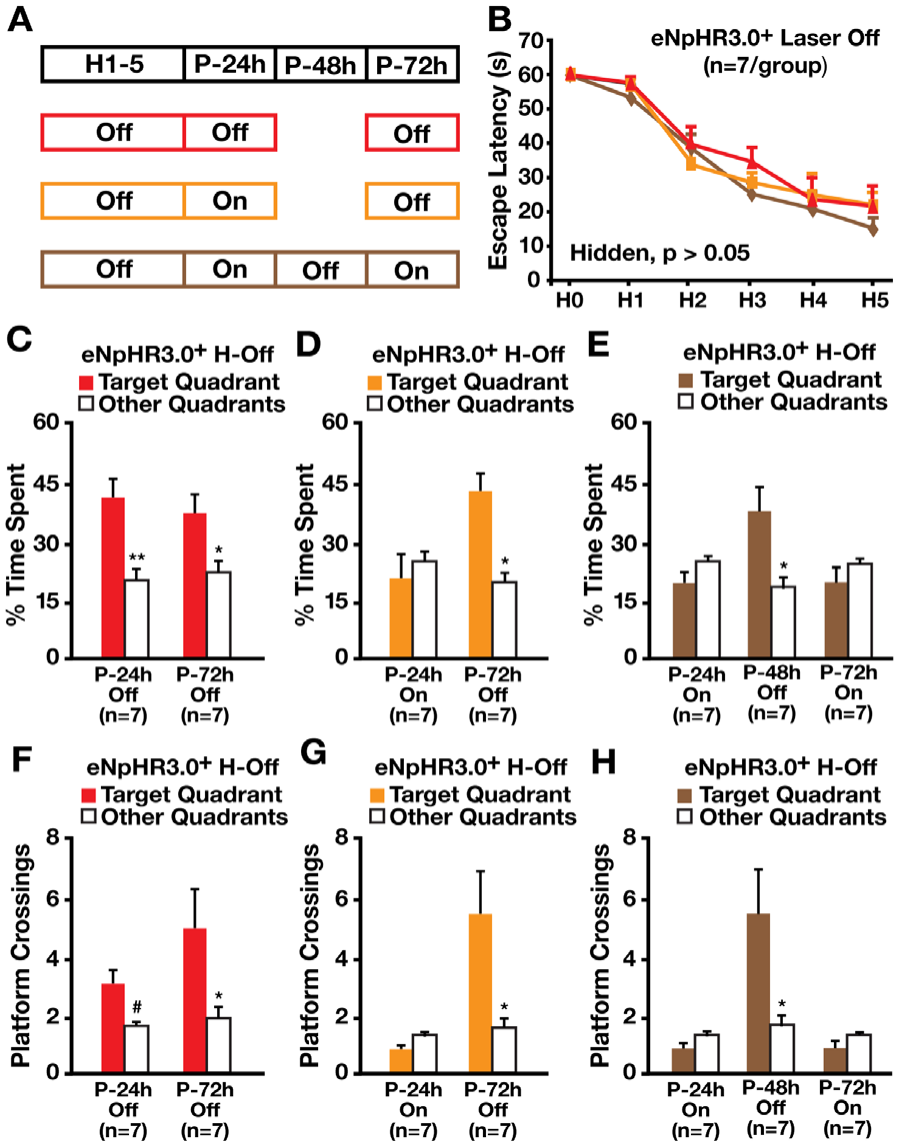
Inhibition of hilar GABAergic interneuron activity impaired spatial memory retrieval but not memory retention. (A) Protocols of mice used and laser illumination during hidden platform (H1–5) and probe (P-24 h, P-48 h, P-72 h) trials in the MWM test. (B) Learning curves of I12b-Cre mice injected with AAV1-DIO-eNpHR3.0-eYFP virus without laser illumination in the hidden platform trials (H-Off) of the MWM test. Points represent averages of daily trials. H, hidden platform sessions (two trials/session, two sessions/day); H0, first trial on H1. Y-axis indicates time to reach the target platform (escape latency). Values are mean ± SEM and statistically evaluated by repeated measures ANOVA. (C–E) Percent time spent in the target quadrant versus the other quadrants in the probe trial performed 24 (P-24 h), 48 (P-48 h), or 72 (P-72 h) hours after the last hidden platform trial with (On) or without (Off) laser illumination. Values are mean ± SEM. *p<0.05, **p<0.01 (two tailed and unpaired *t*-test). F–H, Platform crossings in the probe trial performed 24 (P-24 h), 48 (P-48 h), or 72 (P-72 h) hours after the last hidden platform trial with (On) or without (Off) laser illumination. Values are mean ± SEM. n = 8 mice/group. *p<0.05, ^#^p = 0.05 (two tailed and unpaired *t*-test).

We next determined whether inhibiting hilar GABAergic interneuron activity impairs spatial memory retention, retrieval or both. I12b-Cre mice receiving bilateral hilar injection of AAV1-DIO-eNpHR3.0-eYFP virus were randomly divided into three groups and trained during hidden platform trials without illumination (Fig. 6*A*). All three groups of mice learned equally well during the hidden platform trials (Fig. 6*B*). The first group was tested for spatial memory without laser illumination in probe trials 24 and 72 hours after the last hidden platform trial and showed normal memory for time spent in the target quadrant and platform crossing (Figs. 6*C,F*). The second group, tested for spatial memory with laser illumination in a probe trial 24 hours after the last hidden platform trial, showed memory impairment (Figs. 6*D,G*). Interestingly, the same group had normal memory without illumination at 72 hours (Figs. 6*D,G*), suggesting normal spatial memory retention. Thus, optogenetically inhibiting hilar GABAergic interneuron activity impaired spatial memory retrieval but not memory retention.

**Figure 7:**
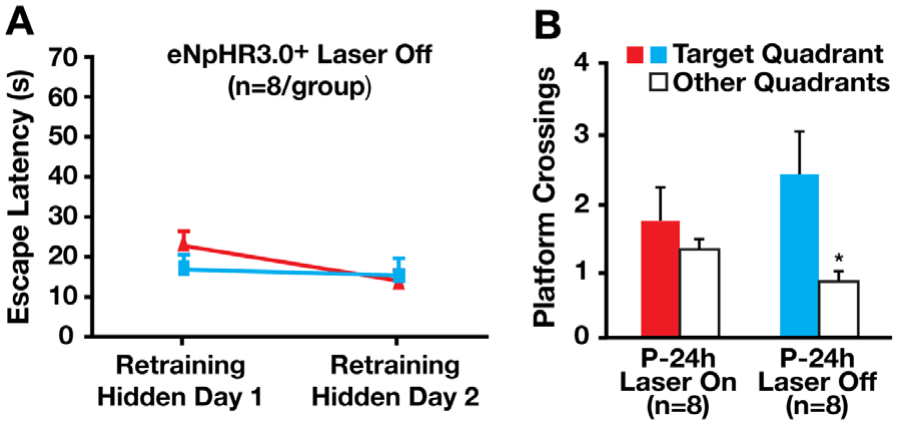
Inhibition of hilar GABAergic interneuron activity impaired spatial memory retrieval in retrained mice. (A) Mice used in Fig. 4 were retrained in the hidden platform trials 2 weeks after the first Morris water maze (MWM) test (see Fig. 4) and showed very good spatial memory. Points represent averages of daily trials. Y-axis indicates time to reach the target platform (escape latency). Values are mean ± SEM. (B) Platform crossings in the probe trial performed with (On) or without (Off) laser illumination 24 h after the last hidden platform trial of the retraining. Values are mean ± SEM. n = 8 mice/group. *p<0.05 (two-tailed and unpaired *t*-test).

To further evaluate this possibility, the third group was tested for spatial memory with illumination at 24 hours, without illumination at 48 hours, and with illumination at 72 hours (Fig. 6*A*). Strikingly, memory was impaired at 24, normal at 48, and impaired again at 72 hours (Figs. 6*E,H*). More strikingly, the same mice retrained 2 weeks after the probe trials located the hidden platform quickly (<22 seconds) in retraining days 1 and 2 (Fig. 7*A*), confirming their normal long-term memory retention. However, bilateral laser illumination impaired memory retrieval again in a probe trial 24 hours later, whereas non-illuminated mice had normal memory retrieval (Fig. 7*B*). These data confirm that inhibiting hilar GABAergic interneuron activity impairs spatial memory retrieval but not memory retention.

### *In vivo* inhibition of hilar GABAergic interneuron activity did not alter short-term working memory, motor coordination, or exploratory activity of mice

**Figure 8:**
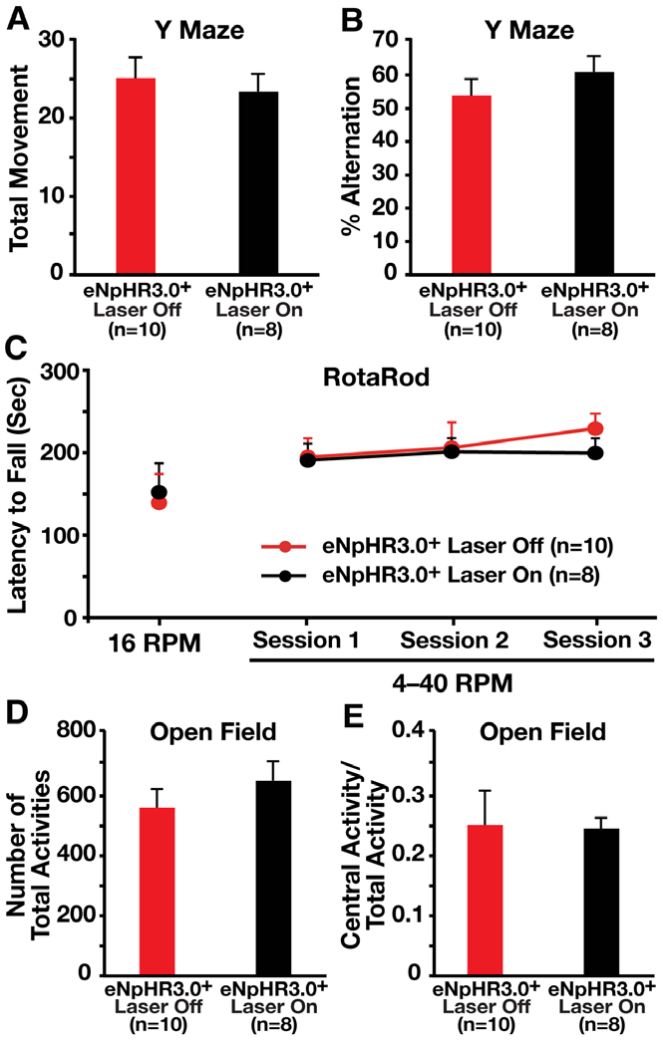
Inhibition of hilar GABAergic interneuron activity did not alter non-hippocampus-dependent behavior. (A, B) I12b-Cre mice injected with AAV1-DIO-eNpHR3.0-eYFP virus were tested for short-term working memory with (On) or without (Off) laser illumination in a Y maze test. Data were reported as total movement (A) and % alternation (B). Values are mean ± SEM. (C) I12b-Cre mice injected with AAV1-DIO-eNpHR3.0-eYFP virus were tested for motor coordination with (On) or without (Off) laser illumination in a rotarod test. Values are mean ± SEM. (D, E) I12b-Cre mice injected with AAV1-DIO-eNpHR3.0-eYFP virus were tested for exploratory activity with (On) or without (Off) laser illumination in a open field test. Data were reported as the number of total activities (D) and the ratio of central activity to total activity (E). Values are mean ± SEM. n = 8–10 mice/group.

Finally, we determined if optogenetic inhibition of hilar GABAergic interneurons alters other behavioral parameters that are not primarily dependent on hippocampal functions. Bilateral illumination of eNpHR3.0^+^ mice did not impair the short-term working memory in a Y-maze test (Figs. 8*A,B*), nor did it alter the motor coordination, as determined by a rotarod test (Fig. 8*C*), or the exploratory activity, as determined in an open field test (Figs. 8*D,E*).

## Discussion

Our data demonstrate that inhibiting hilar GABAergic interneuron activity impairs spatial learning and memory retrieval, without affecting spatial memory retention or short-term working memory. In line with this conclusion, learning triggers a rapid increase in inhibitory synaptogenesis and the GABA content of inhibitory synapses [20], accompanied by long-lasting enhancement of synaptic inhibition onto excitatory neurons in mice [21]. Learning also triggers a lasting increase in GABA release from hippocampal GABAergic interneurons in mice [6], [22], and learning-related feed-forward inhibitory connectivity growth in the hippocampus is required for memory precision [23]. Conversely, decreasing GABA levels in the hippocampus by overexpressing GABA transport 1 (GAT1), which is responsible for GABA reuptake after its synaptic release, impairs learning and memory in mice [24]. Thus, learning appears to involve an increase in inhibitory synaptic plasticity and GABA release. Interestingly, it has been reported that parvalbumin-positive GABAergic interneurons in the CA1 region of the hippocampus are required for working memory but not for reference memory [25]. Thus, GABAergic interneurons in different hippocampal subregions may control different types of memory.

The hippocampus has historically been viewed as a temporary memory structure for retention and retrieval of long-term memories. These memories were thought to eventually become independent of the hippocampus as they become consolidated in extra-hippocampal structures, such as the neocortex, where they are stored and available for retrieval without hippocampal involvement [26], [27], [28], [29], [30], [31]. Pioneering work on the process of contextual fear memory consolidation showed that hippocampal lesions impaired recent memories one day after training, but the same lesions had no effect on remote memory several weeks after training [26], [27], [29]. Studies on human patients with medial temporal lobe injuries suggested a similar conclusion, where patients exhibited a temporally graded retrograde amnesia in which information acquired shortly before surgery was lost whereas older memories were retained [28], [32]. However, in recent years, experimental findings in both the human and the animal literature are in conflict with this original theory. There are many cases of patients with memory loss after medial temporal amnesia with no temporal gradient, and it has been shown that the hippocampus may not only be involved in encoding, but may also contribute to storage and retrieval of memory [33], [34]. Recent animal studies also showed that hippocampal memory was not merely replaced by the cortical one, but rather both memories are in continuous interplay and there may indeed be a default role for the hippocampus in remote memory recall [33], [35], [36], [37], [38]. Intriguing recent studies have shown a default role for the hippocampus in remote memory recall [37], [39], including a study where optogenetic inhibition of the CA1 pyramidal neurons in the hippocampus was sufficient to impair remote recall of memories using the contextual fear conditioning learning paradigm [38]. In agreement with these recent developments in the understanding of the role of the hippocampus in long term memory, we observed that precise, real–time inhibition of GABAergic interneuron activity in the hilus of the hippocampus, using optogenetic techniques, impairs memory retrieval up to 2 weeks after the initial memory formation, highlighting the importance of the hilar inhibitory interneurons in long-term memory retrieval.

There is evidence that GABAergic interneuron impairment might be involved in the pathogenesis of AD. These include reduced somatostatin immunoreactivity in the cerebral cortex from cases of AD [40] and a decrease in the cerebrospinal fluid concentrations of GABA in AD patients [41], [42], [43], [44]. The loss of somatostatin immunoreactivity in AD brains is exacerbated by the presence of apolipoprotein (apo) E4, the major known genetic risk factor for AD [45]. Our findings support the potential contribution of GABAergic interneuron impairment to the pathogenesis of amnesia in AD. At early stages of AD, patients usually experience fluctuations between accurate memory and memory lapse, or amnesia, of an old event, suggesting that their memory retention is largely intact, but memory retrieval is periodically and reversibly impaired, similar to the phenotype of mice with transient optogenetic inhibition of hilar GABAergic interneuron activity. Thus, GABAergic interneuron impairment might contribute to the pathogenesis of the fluctuating amnesia at the early stage of AD.

Furthermore, transgenic mice expressing amyloid precursor protein with AD-related mutations, which develop spatial learning and memory deficits [46], have impaired hilar GABAergic interneuron function, leading to overexcitation in the dentate gyrus [46], [47]. Knock-in mice expressing the major AD genetic risk factor apoE4 [48], which also develop spatial learning and memory deficits, have age-dependent loss of hilar GABAergic interneurons, leading to reduced inhibition of dentate granule neurons [7]. Since the mutant amyloid precursor protein or apoE4 caused multiple neuronal deficits in addition to hilar GABAergic interneuron impairment [8], [49], these transgenic or knock-in mouse studies failed to establish a causal relationship between hilar GABAergic interneuron impairment and spatial learning and memory deficits. Our study illustrates the essential value of optogenetic control in dissecting the roles of different neuronal populations in different brain regions in learning and memory formation, and demonstrates precisely that impairing hilar GABAergic interneuron function alone causes spatial learning and memory deficits. Consequently, drugs that enhance hilar GABAergic interneuron function might be beneficial for treating amnesia in AD and other amnesia-related neurological conditions. In fact, treatment of apoE4 knock-in mice with the GABA_A_ receptor potentiator pentobarbital prevents apoE4-caused spatial learning and memory deficits [7].
